# Korean Medication Algorithm for Bipolar Disorder: changes in preferred medications for mania over 20 years

**DOI:** 10.1192/j.eurpsy.2024.534

**Published:** 2024-08-27

**Authors:** D.-I. Jon, Y. S. Woo, J.-H. Jeong, J.-S. Seo, J. G. Lee, B.-H. Yoon, I. Sohn, W.-M. Bahk

**Affiliations:** ^1^Hallym University Sacred Heart Hospital, Anyang; ^2^The Catholic University; ^3^Joongang University, Seoul; ^4^Inje University, Busan; ^5^Naju National Hospital, Naju; ^6^Kyeyo Hospital, Euwang, Korea, Republic Of

## Abstract

**Introduction:**

Majority of international guidelines for bipolar disorders are based on evidences from clinical trials. In contrast, the Korean Medication Algorithm Project for Bipolar Disorder (KMAP-BP) was developed to adopt an expert-consensus paradigm which was more practical and specific to the atmosphere in Korea.

**Objectives:**

In this study, preferred medication strategies for acute mania over six consecutively published KMAP-BP (2002, 2006, 2010, 2014, 2018, and 2022) were investigated.

**Methods:**

A written survey using a nine-point scale was asked to Korean experts about the appropriateness of various treatment strategies and treatment agents. A written survey asked about the appropriateness of various treatment strategies and treatment agents commonly used by clinicians as the first-line.

**Results:**

The most preferred option for the initial treatment of mania was a combination of a mood stabilizer (MS) and an atypical antipsychotic (AAP) in every edition. Preference for combined treatment for euphoric mania increased, peaked in KMAP-BP 2010, and declined slightly. Either MS or AAP monotherapy was also considered a first-line strategy for mania, but not for all types of episodes, including mixed/psychotic mania. Among MSs, lithium and valproate are almost equally preferred except in the mixed subtype where valproate is the most recommended MS. The preference of valproate showed reverse U-shaped curve. This preference change of valproate may indicate the concern about teratotoxicity in women. Quetiapine, aripiprazole, and olanzapine were the preferred AAP for acute mania since 2014. This change might depend on the recent evidences and safety profile. In cases of unsatisfactory response to initial medications, switching or adding another first-line agent was recommended. The most notable changes over time included the increasing preference for AAPs.

**Conclusions:**

The Korean experts have been increasingly convinced of the effectiveness of a combination therapy for acute mania. There have been evident preference changes: increased for AAP and decreased for carbamazepine.

**Disclosure of Interest:**

None Declared

